# Isolated Proximal Tibiofibular Dislocation during Soccer

**DOI:** 10.1155/2015/657581

**Published:** 2015-12-02

**Authors:** Casey Chiu, Johnathan Michael Sheele

**Affiliations:** Department of Emergency Medicine, University Hospitals Case Medical Center and Case Western Reserve University, Cleveland, OH 44106, USA

## Abstract

Proximal tibiofibular dislocations are rarely encountered in the Emergency Department (ED). We present a case involving a man presenting to the ED with left knee pain after making a sharp left turn on the soccer field. His physical exam was only remarkable for tenderness over the lateral fibular head. His X-rays showed subtle abnormalities of the tibiofibular joint. The dislocation was reduced and the patient was discharged from the ED with orthopedic follow-up.

## 1. Introduction

Isolated dislocation of the proximal tibiofibular joint is an uncommon injury but has been associated with multiple different sports injuries including parachuting, ballet, and rugby as well as motor vehicle accidents [1, 2]. Anterolateral dislocations are the most common tibiofibular joint dislocation and are seen in 85% of cases [3]. The mechanism leading to a tibiofibular joint dislocation usually involves a sudden internal rotation and plantar flexion of the foot and an external rotation of the leg with flexion of the knee or a fall on a flexed adducted leg [3].

The proximal tibiofibular joint is a synovial joint between the oval facet of the head of the fibula and the facet of the lateral tibial condyle. The anterior and posterior tibiofibular ligaments and the joint capsule provide stabilization for the joint [1]. The clinical signs of a tibiofibular joint dislocation include swelling and tenderness around the tibiofibular joint and the proximal fibula. Patients can present with a sensation that the knee feels “out of joint [1].”

Plain radiographs are helpful for diagnosing tibiofibular joint dislocations. The X-rays may show a laterally displaced fibular head and a widened interosseous space. If the diagnosis is uncertain, then X-rays of the unaffected contralateral tibiofibular joint can be helpful in making the diagnosis [1].

## 2. Case Presentation

A 21-year-old male soccer player with no history of prior knee injuries presents to the ED via private vehicle complaining of left proximal leg pain and difficulty ambulating. The patient comes to the ED directly from the soccer field where he states he was running and then made an abrupt cut to the left causing his ankle to roll. He then heard a crack from his knee and immediately felt pain along the lateral side of his proximal leg and proceeded to fall. The patient denied any pain while flexing or extending his knee. The patient states that he may have adducted his left leg while in a flexed position when he cut to the left. The patient complained of his left knee feeling “tight.” The patient had no other injuries or complaints and the rest of his physical exam was unremarkable.

**Figure 1:**
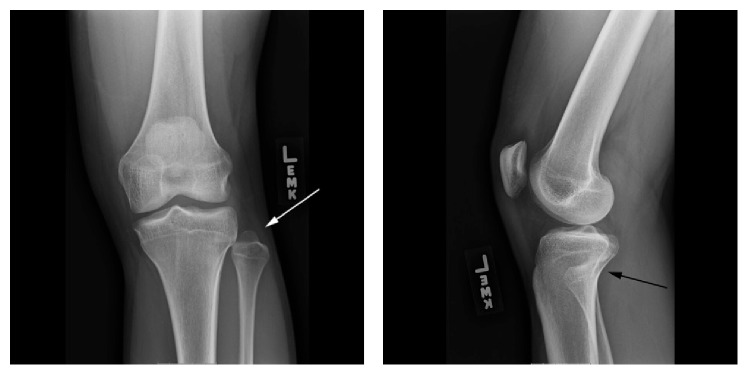
AP and lateral X-ray views of the left knee showing Type II anterolateral tibiofibular dislocation.

**Figure 2:**
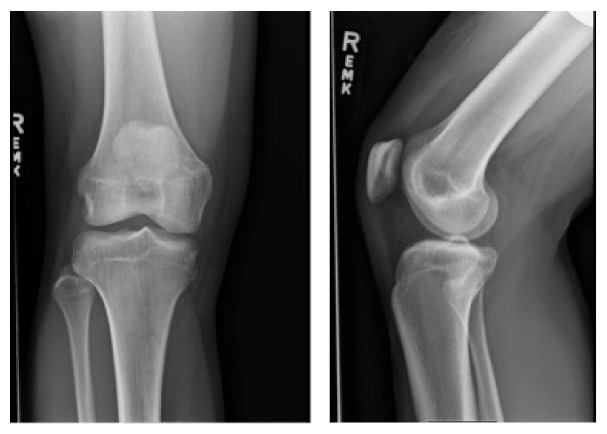
AP and lateral X-ray views of the right “unaffected” knee.

**Figure 3:**
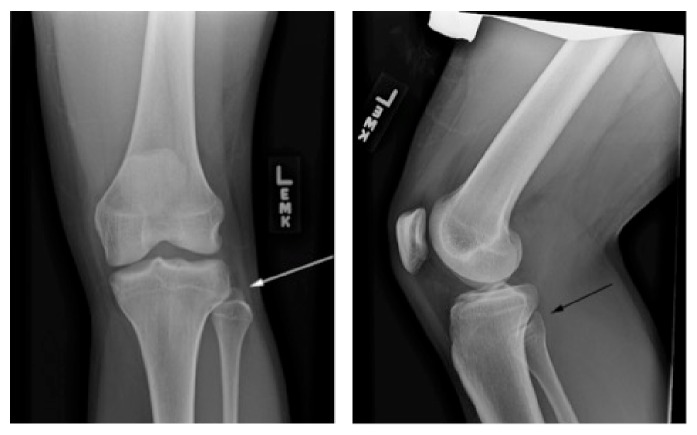
AP and lateral X-ray views of the left knee after reduction of the tibiofibular dislocation.

On examination, the patient appeared in no distress and his vital signs were stable. The patient had 5/5 strength with dorsiflexion and plantarflexion. Both knee joints were stable with Lachman, posterior drawer, varus stress, and valgus stress test. Just by visual inspection, the left fibular head appeared more pronounced when compared to the contralateral side. The patient also complained of severe tenderness upon palpation over the fibular head with varus stress of the left knee. There was no crepitus to flexion or extension of the left knee. Anterior-posterior (AP) and lateral view X-rays of the left knee are shown in Figure 1.  Figure 2 shows the contralateral AP and lateral X-ray views of the unaffected right knee. The AP view of the affected left knee shows that the fibular head was situated with a more lateral prominence and with less tibia-fibula overlap compared to the right knee. On the lateral views, the left fibular head is very slightly more anterior compared to the contralateral knee. The constellation of history, physical examination, and X-ray finding suggested that the patient had a proximal anterolateral fibular head dislocation. Closed reduction of the left fibular head dislocation was performed by orthopedics. Immediately after joint relocation the patient stated that the left knee pain had improved and the “tightness” had resolved.  Figure 3 shows post-X-ray reduction films of the left knee and normal anatomical alignment of the left tibiofibular joint. The patient was placed in a knee immobilizer, given crutches, and referred to orthopedics.

## 3. Discussion

A tibiofibular dislocation is a clinical diagnosis that can often be misdiagnosed as a meniscal injury. If untreated, the patient may experience chronic pain, abnormal gait, and reduced sports performance [4]. A high index of suspicion for a tibiofibular joint dislocation needs to be maintained in all patients who present with lateral knee pain and an inability to bear weight.

There are four types of tibiofibular joint dislocations [2]. Type I is a subluxation of the proximal tibiofibular joint [2]. Type II, the most common type of dislocation, is an anterior dislocation that involves the anterior and posterior tibiofibular ligaments [2]. Type III dislocation involves a posteromedial dislocation that can rarely injure the peroneal nerve [2]. Type IV dislocation involves a superior dislocation and is associated with a tibial shaft fracture or ankle injury [2].

The physical examination for a tibiofibular joint dislocation may only show subtle findings such as a prominent fibular head that can be accentuated by knee flexion with an anterior dislocation [5]. The patient with a tibiofibular joint dislocation often presents with lateral knee pain, a normal range of motion of the affected knee, and no joint effusion [6, 7]. Patients with chronic tibiofibular joint dislocations may have a “popping” sensation in the knee and will often complain of maximal tenderness over the fibular head.

Obtaining X-rays of the AP and lateral views of the unaffected knee for comparison to the painful knee may suggest an abnormality at the tibiofibular joint. Alternatively a computed tomography (CT scan) or magnetic resonance imaging (MRI) can be used to make the diagnosis [8].

Tibiofibular joint dislocations need to be reduced, usually with the knee in flexion, and a “pop” may be heard or felt as the joint is reduced [7]. Patients are then instructed to remain non-weight-bearing until appropriate follow-up with orthopedics [2]. Peroneal nerve injury can occur in up to 5% of tibiofibular dislocations and patients may be more prone to degenerative joint disease in the future [5].

Our patient dislocated his tibiofibular joint after a twisting motion of his left knee. He presented to the ED with a prominent and painful fibular head. The patient had appropriate range of motion and normal laxity of the knee. Reduction of the dislocated joint was accomplished by direct manipulation. In summary, we report a rare case of a tibiofibular joint dislocation and we review the diagnosis and management of the condition. Suspicion for tibiofibular dislocation is needed for persons presenting to the ED with lateral knee pain after a fall and a twisting of the knee.
